# Surface Adsorption of the Cancer Biomarker Lysophosphatidic Acid in Serum Studied by Acoustic Wave Biosensor

**DOI:** 10.3390/ma14154158

**Published:** 2021-07-27

**Authors:** Brian De La Franier, Michael Thompson

**Affiliations:** Department of Chemistry, University of Toronto, 80 St. George Street, Toronto, ON M5S 3H6, Canada; brian.delafranier@mail.utoronto.ca

**Keywords:** lysophosphatidic acid, surface-modified gold, acoustic wave biosensor, non-specific adsorption

## Abstract

The thickness shear mode acoustic wave device is of interest for the sensing of biomarkers for diseases in various biological fluids, but suffers from the issue of non-specific adsorption of compounds other than those of interest to the electrode surface, thus affecting the device’s output. The aim of this present study was to determine the level of non-specific adsorption on gold electrodes from serum samples with added ovarian cancer biomarker lysophosphatidic acid in the presence of a surface anti-fouling layer. The latter was an oligoethylene molecule with thiol group for attachment to the electrode surface. It was found that the anti-fouling layer had a minimal effect on the level of both adsorption of components from serum and the marker. This result stands in sharp contrast to the analogous monolayer employed for anti-fouling reduction on silica.

## 1. Introduction

The thickness shear mode (TSM) acoustic wave biosensor, sometimes referred to as a “quartz crystal microbalance”, is typically composed of a pair of gold electrodes placed on both sides of a thin disc of piezoelectric quartz. When an alternating electric field is applied, a mechanical oscillation involving a standing wave is established across the bulk of the quartz wafer [1,2]. Sensing can be achieved by binding a probe molecule to the surface of the gold substrate, which binds selectively to the analyte of interest, causing a measurable change in vibrational frequency and motional resistance. However, due to the complex nature of bio fluids, non-specific adsorption or fouling of compounds on the electrode surface, other than those of interest, renders these devices difficult to use in these fluids [3]. For example, serum proteins, such as human serum albumin and oligopeptides, can attach to gold via sulfhydryl groups [4,5].

A vast literature describes the many attempts that have been made to avoid the fouling of surfaces by components of biological fluids [6]; this includes gold as a substrate. Given the preponderance of thiol or disulfide chemistry on Au over recent years, it is unsurprising that a variety of sulfur-containing compounds, including Zwitterionic and other species formed into self-assembled monolayers (SAMS), have been used to reduce non-specific adsorption on Au with a level of success [7,8,9,10]. In our own work, we have used 2-(3-trichlorosilylpropyloxy)-ethyl hydroxide (MEG-OH) as an anti-fouling layer on a variety of materials, and it has been found to reduce fouling from serum, whole blood, and bacteria [11,12,13,14,15,16,17]. The layer formed by this compound is made up of a siloxane network that enables water molecules to penetrate and hydrogen bond to the inner ether groups present, which creates an interstitial hydration layer that acts to anti-foul the surface [18,19].

**Figure 1:**
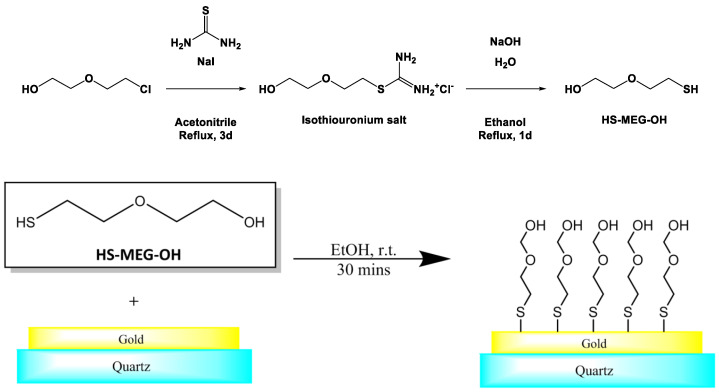
Synthesis and self-assembly of 2-(2-mercaptoethoxy)ethan-1-ol to gold electrodes of a TSM device.

We have used custom-synthesized 2-(2-mercaptoethoxy)ethan-1-ol (HS-MEG-OH) to investigate the key role of surface hydration on the dynamics of adsorption of components of serum to gold [20] (Figure 1). In order to further evaluate the anti-fouling capability of HS-MEG-OH layers on TSM electrodes, the surface chemistry of the cancer biomarker lysophosphatidic acid (LPA) was chosen for this purpose. LPA is a potential marker for ovarian cancer which has been found to be elevated in 90% of stage I ovarian cancer patients, and 100% of later-stage patients [21,22,23,24]. If the TSM is to be employed as an early-stage detection platform for LPA in serum, via a device-anchored probe for the marker, the avoidance of fouling will be mandatory. However, as the thiol link required to bind this molecule to gold does form a siloxane network, it is unlikely that the layer will present adequate spacing to allow water to form the interstitial hydration layer. Thus, it is unlikely to prevent fouling to the extent that MEG-OH does.

## 2. Materials and Methods

### 2.1. Materials

Sodium dodecyl sulfate, phosphate-buffered saline (PBS) buffer, and goat serum were all purchased from Sigma-Aldrich, Oakville, ON, Canada. Lysophosphatidic acid was obtained from Enzo Life Sciences via FroggaBio Inc., ON, Canada and Nitrogen as a pressurized gas from Praxair, ON, Canada. Quartz crystals (AT-cut, 13.5 mm in diameter, 9 MHz fundamental frequency) with gold electrodes in place were purchased from Lap-Tech Inc. Bowmanville, ON, Canada. The devices were systematically handled with thoroughly pre-cleaned stainless steel tweezers in order to minimize any external contamination. The synthesis of 2-(2-mercaptoethoxy)-ethan-1-ol (HS-MEG-OH) has been described elsewhere [20].

### 2.2. Surface Modification of TSM Gold Electrodes

Quartz discs were placed in small glass vials, which had been rinsed 2 times with deionized water and once with 1% sodium dodecyl sulfate (SDS) solution. Each disc was then rinsed 2 times with SDS solution followed by placement on a rotator in the same solution for 15 min. Subsequent to a thorough rinsing in water, the devices were rinsed 3 times in acetone. After that, they were rinsed 3 times with methanol, followed by 5 min of rotation in the same matrix. They were then rinsed thoroughly with methanol, and dried under a stream of nitrogen. Finally, the discs were plasma-cleaned for 5 min, followed by storage in glass vials.

Cleaned TSM discs were soaked in 5 µM HS-MEG-OH in anhydrous absolute ethanol, and placed on a rotator for 30 min. The discs were then rinsed with absolute ethanol, followed by methanol and dried under a stream of nitrogen. Finally, the discs were stored in glass vials.

### 2.3. TSM Operation and Measurements

A TSM disc was placed in a custom flow-through chamber connected to a Maxtek PLO-10i oscillator (Inficon, East Syracuse, NY, USA). A Harvard Apparatus (Holliston, MA, USA) 11plus syringe pump was used to flow 0.01 M PBS buffer at pH 7.4 through the chamber and over the disc surface at 100 µL min^−1^. Once the disc was saturated in PBS solution, the PLO-10i oscillator was calibrated using the calibration rotors on the front of the device. Analogue signals from the oscillator were converted into a USB digital signal by way of a custom-designed A/D circuit and Parallax QuickStart board (Parallax, Rocklin, CA, USA) with the data being recorded by custom software programmed in Visual Basic. Once the frequency of the disc had stabilized in PBS, the test solution (such as LPA in PBS, or dilute serum) was passed over the disc for 5 min, before returning the disc to PBS. The disc was kept under PBS flow for 30 min after the test solution was stopped, followed by switching the solution to water for final washing of the disc and chamber.

Data was analyzed by taking the average frequency of the TSM discs in PBS for 10 min prior to injection of the sample. The frequency change when in a particular sample was averaged for 1.5 min, 5 min after injection. Finally, the return frequency was averaged from 20–30 min after return to the PBS solution for washing. Calculations and graphing were performed in Microsoft Excel. At least 3 runs were averaged for each solution and the errors shown are the standard deviations from multiple runs. With respect to motional resistance values, the PLO-10i interface outputs the overall capacitance of the device as a voltage, which must then be converted to a resistance value. This was achieved by dividing 100 by the capacitance and subtracting 20 (the system’s internal resistance), which gives a value for the resistance of the TSM system (Equation (1)).

(1) $$R=\frac{100}{C}-20$$

**Equation (1)**. Calculation for crystal resistance (*R*) based on the instrument capacitance (*C*).

## 3. Results

### 3.1. Resonant Frequency Responses

**Figure 2:**
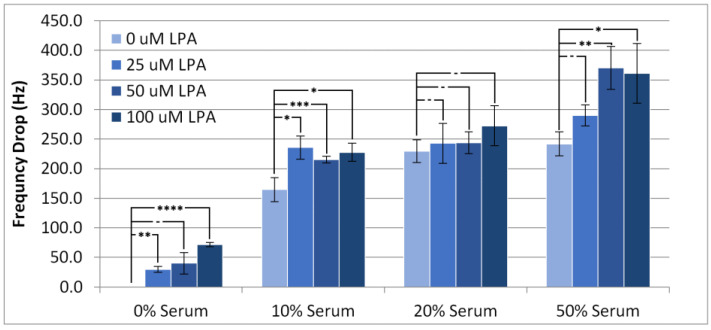
Frequency drop of the TSM signal on addition of PBS/serum samples containing LPA. Statistical significance using *t*-test shown by asterisks: (-) no significant difference, (*) 90% confidence, (**) 95% confidence, (***) 98% confidence.

Individual experimental responses in TSM frequency and motional resistance over time can be found in the Supporting Information (Figures S1–S8). Figure 2 shows the lowest frequency of the TSM with the surface modifier in place after injection of serum samples at different concentrations of LPA and serum (diluted with PBS buffer solution). This response is conventionally referred to as the frequency drop. As anticipated, the frequency drop was lowest when there was no serum present, although there was statistically minimal change in the frequency drop between 10% and 50% serum when LPA was present, which suggests that the amount of material deposited onto the device is invariable over this concentration range. Notably, the addition of LPA into the injection solution tended to increase the frequency drop in all conditions with or without the presence of serum, except in the case of 20% serum. This result strongly indicates that the HS-MEG-OH layer does not prevent either the non-specific adsorption of components of serum or LPA itself.

At 10% serum concentration, there was no difference in frequency drop for any addition of LPA, but all LPA-doped samples were different from just serum. When the serum concentration was increased to 20%, there was no significant effect of LPA concentration on the frequency drop with all conditions exhibiting frequency drops of similar value. At 50% serum, the increase in drop at 25 µM LPA is not statistically different from no LPA, but 50 µM LPA and 100 µM LPA show a slight difference from no LPA. This suggests that at 10% and 50% serum, LPA has an effect on the deposition of materials onto gold, which saturates between 25 µM and 50 µM LPA, an effect which is not present at 20% serum.

**Figure 3:**
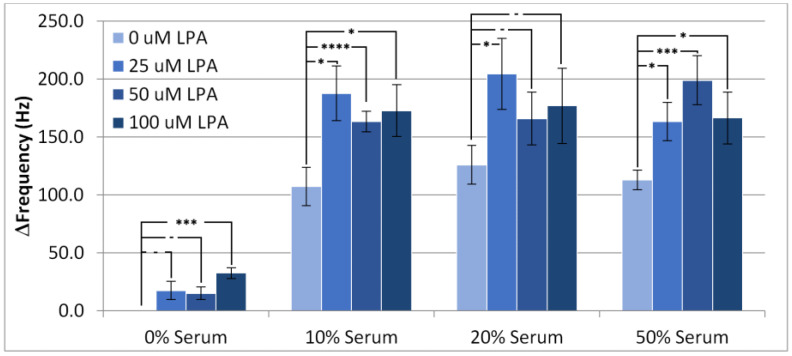
Change in frequency of the TSM from the baseline in PBS buffer to the final wash for experiments conducted with PBS/serum solutions containing LPA. Statistical significance using *t*-test shown by asterisks: (-) no significant difference, (*) 90% confidence, (***) 98% confidence, (****) 99% confidence.

The change in frequency from before injection to after wash-off with PBS, which is referred to as the overall frequency change, is depicted in Figure 3. These experiments clearly indicate the amount of “permanently” adsorbed material retained by the surface. From the samples with no additional LPA, it can be seen that all three serum concentrations exhibit a similar amount of overall frequency change, or fouling. This indicates that increasing serum concentrations from 10% to 50% has little impact on the amount of non-specific adsorption. Accordingly, with the surface modifier in place, the interface reached saturation at the lower level of 10%. The addition of LPA to the samples increased the overall fouling compared to serum samples containing no LPA. This suggests that the adlayer reduces fouling from serum components to some degree, as LPA is able to foul beyond these serum components.

However, a significant level of change in frequency still occurred for all serum concentrations, suggesting that HS-MEG-OH is unable to prevent fouling of the surface to the extent that MEG-OH is able to. As the silane version of MEG-OH has been found to provide significant anti-fouling in similar acoustic wave biosensors [11,16], this suggests that the spacing provided between each monoethylene-glycol group, by the siloxane network of MEG-OH and the resulting surface hydration, is very important to the layer’s anti-fouling ability. As the thiol links of HS-MEG-OH do not provide such spacing, it seems that the hydration layer is unable to form, and higher levels of fouling are observed compared to MEG-OH. As this is the only structural difference between the layers, this is the most likely reason for the observed inability for HS-MEG-OH to prevent fouling, lending evidence to previous work establishing the mechanism of action for MEG-OH, which had been performed using neutron reflectometry as well as computer simulation [18,19].

In order to solve this issue on gold and allow for a similar ultrathin anti-fouling layer to be produced, different monoethylene glycol thiol-based layers that exhibit spacing should be explored, such as using dithiol linking groups to increase the space between the glycol molecules. This will allow for further determination of the importance of the hydration layer should a better-spaced layer prove to be capable of antifouling, similar to that observed for the silane version.

### 3.2. Responses in Resistance

**Figure 4:**
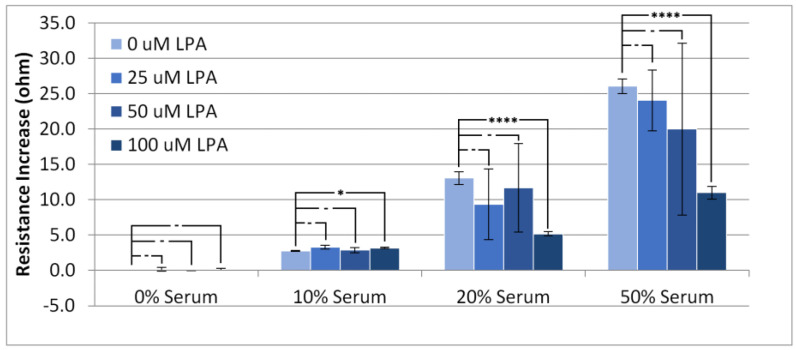
Resistance increase in the TSM signal on addition of PBS/serum samples with LPA. Statistical significance using *t*-test shown by asterisks: (-) no significant difference, (*) 90% confidence, (****) 99% confidence.

**Figure 5:**
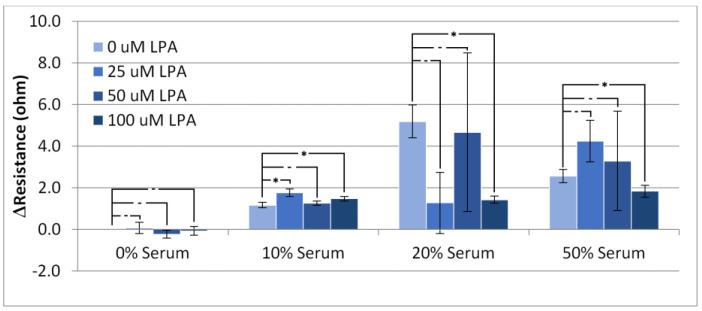
Overall change in resistance of the TSM between the initial resistance in PBS buffer and the resistance following the final wash. Statistical significance using *t*-test shown by asterisks: (-) no significant difference, (*) 90% confidence.

Figure 4 shows, as expected, that increasing the serum concentration results in higher values for the device resistance. The addition of LPA to the injected solutions seemed to have little effect on the resistance increase for the PBS and 10% serum experiments, whereas at higher concentrations of serum, LPA seemed to cause a large variation in the response with respect to resistance, with 100 µM LPA causing a statistically significant change from no LPA. A similar trend to the rise in resistance can be observed with the overall resistance change (Figure 5). There is little overall change to the resistance of the crystal at low concentrations of serum, and a large overall change can be observed with high concentrations of serum.

As with the resistance rise, LPA seemed to have little effect on the overall change in resistance at low concentrations of serum, but caused large variation in experiments performed at higher concentrations. Taken on its own, the overall resistance change is not useful for analyzing the system due to the high level of exhibited variability. This high level of variability lends further evidence to HS-MEG-OH’s inability to prevent surface fouling observed in the frequency changes. As the resistance change was also quite significant, and highly erratic, it does not lend itself to an alternate form of measurement when using this layer. Unfortunately, for HS-MEG-OH, this suggests that the layer is unable to reduce fouling to a point of being able to usefully measure biomarker concentrations in serum samples. However, as discussed previously, this result lends evidence to the importance of the hydration layer found in similar silane-based layers, and efforts should be undertaken to investigate layers on gold that provide similar spacing to the siloxane network.

## 4. Conclusions

In order to produce a viable biosensor-based technology for the detection of LPA in patient serum, hopefully undiluted, it will be mandatory to avoid non-specific adsorption on the device surface, whether originating from the components of the biological fluid or the biomarker itself. With respect to a typical TSM sensor, this requires modification of the gold surface. Previous research has indicated that the highly successful strategy of modification of silica with the analogous trichlorosilane MEG-OH [16] is not reproduced with the sister molecule HS-MEG-OH [20], and this result appears to be confirmed in the present work on gold-serum-LPA surface chemistry. On silica, the significant reduction in fouling can be attributed to the presence of interstitial hydration of the surface monolayer, which has either an energy cost in terms of disturbance (so-called water barrier effect) or no energy gain from the interaction of hydrated species with the water-containing monolayer [25,26]. In the case of the surface modifier described in the present work, the thiol groups will cause relatively close packing in molecular terms, so that the formation of the key hydration layer is not feasible. This negative result strongly suggests that if the TSM is to be employed for biomarker detection in biological fluids, an alternative strategy for the avoidance of fouling must be invoked. In order to better simulate the anti-fouling ability of MEG-OH on gold sensor surfaces, an anti-fouling layer that provides sufficient spacing for the hydration layer to form should be investigated, such as one based on a dithiol linker or possibly using a bulky side group below the monoethylene glycol to encourage spacing between individual molecules.

## Data Availability

Data is contained within the article or supplementary material.

## Supplementary Materials

The following are available online at https://www.mdpi.com/article/10.3390/ma14154158/s1, Figure S1: Responses in TSM frequency for 100% PBS buffer containing (**a**) 0 μM LPA, (**b**) 25 μM LPA, (**c**) 50 μM LPA, and (**d**) 100 μM LPA; Figure S2: Responses in TSM frequency for PBS buffer with 10% serum containing (**a**) 0 μM LPA, (**b**) 25 μM LPA, (**c**) 50 μM LPA, and (**d**) 100 μM LPA; Figure S3: Responses in TSM frequency for PBS buffer with 20% serum containing (**a**) 0 μM LPA, (**b**) 25 μM LPA, (**c**) 50 μM LPA, and (**d**) 100 μM LPA; Figure S4: Responses in TSM frequency for PBS buffer with 50% serum containing (**a**) 0 μM LPA, (**b**) 25 μM LPA, (**c**) 50 μM LPA, and (**d**) 100 μM LPA; Figure S5: TSM resistance response for PBS buffer containing (**a**) 0 μM LPA, (**b**) 25 μM LPA, (**c**) 50 μM LPA, and (**d**) 100 μM LPA; Figure S6: TSM resistance response for PBS buffer with 10% serum containing (**a**) 0 μM LPA, (**b**) 25 μM LPA, (**c**) 50 μM LPA, and (**d**) 100 μM LPA; Figure S7; TSM resistance response for PBS buffer with 20% serum containing (**a**) 0 μM LPA, (**b**) 25 μM LPA, (**c**) 50 μM LPA, and (**d**) 100 μM LPA; Figure S8: TSM resistance response for PBS buffer with 50% serum containing (**a**) 0 μM LPA, (**b**) 25 μM LPA, (**c**) 50 μM LPA, and (**d**) 100 μM LPA.
