# Supplementary material for: Surface Adsorption of the Cancer Biomarker Lysophosphatidic Acid in Serum Studied by Acoustic Wave Biosensor
Source: Materials (Basel). 2021 Jul 27;14(15):4158. doi: 10.3390/ma14154158 (PMC8347737; doi:10.3390/ma14154158)
Supplement: Supplementary file 1 [file materials-14-04158-s001.zip › materials-1289604-supplementary.pdf]

materials

MDPI

Supporting information

Surface Adsorption of the Cancer Biomarker Lysophosphatidic Acid in Serum Studied by Acoustic Wave Biosensor

Brian De La Franier and Michael Thompson*

Department of Chemistry, University of Toronto, 80 St. George Street, Toronto, ON M5S 3H6, Canada; brian.delafranier@mail.utoronto.ca
* Correspondence: m.thompson@utoronto.ca; Tel.: +1-416-978-3575

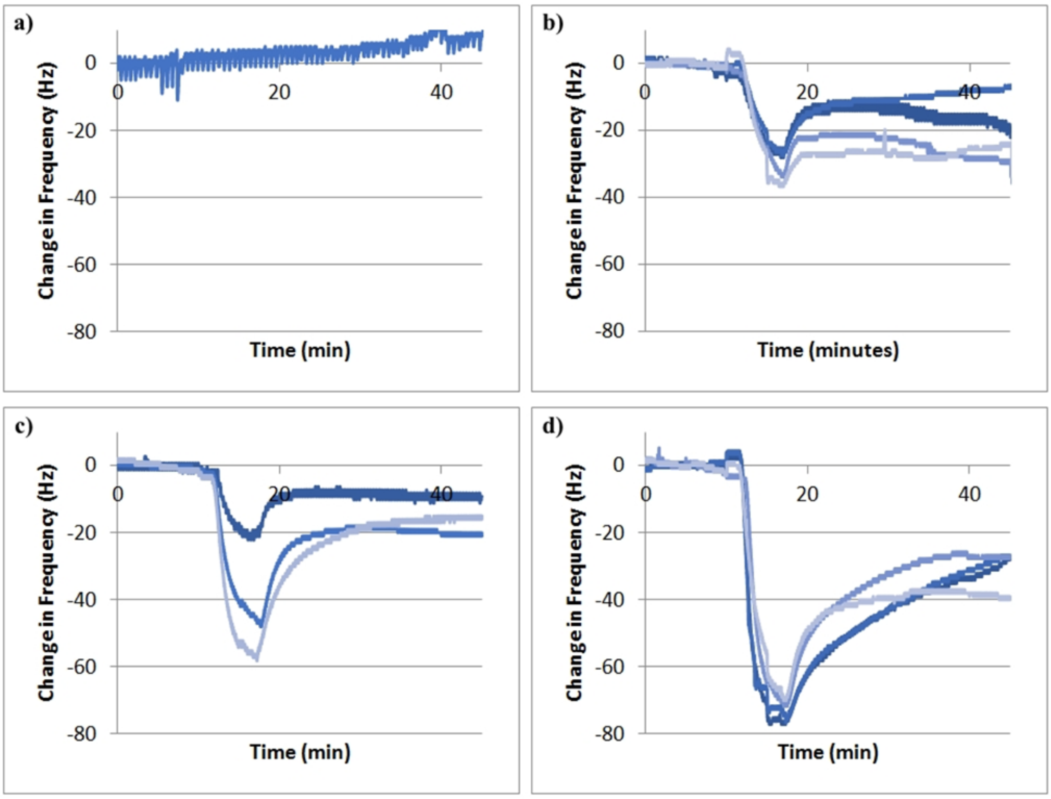

Figure S1. Frequency data for individual TSM runs of 100% PBS buffer containing (**a**) 0 µM LPA, (**b**) 25 µM LPA, (**c**) 50 µM LPA, and (**d**) 100 µM LPA.

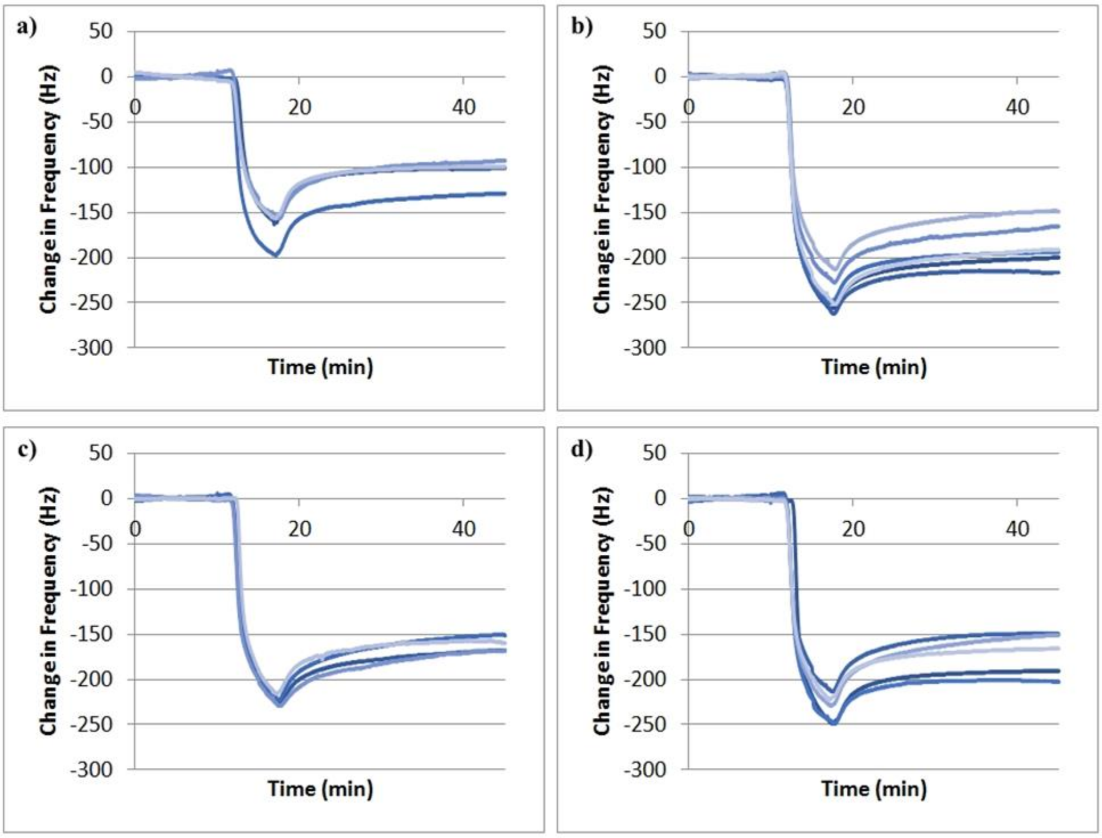

Figure S2. Frequency data for individual TSM runs of PBS buffer with 10% serum containing (**a**) 0 µM LPA, (**b**) 25 µM LPA, (**c**) 50 µM LPA, and (**d**) 100 µM LPA.

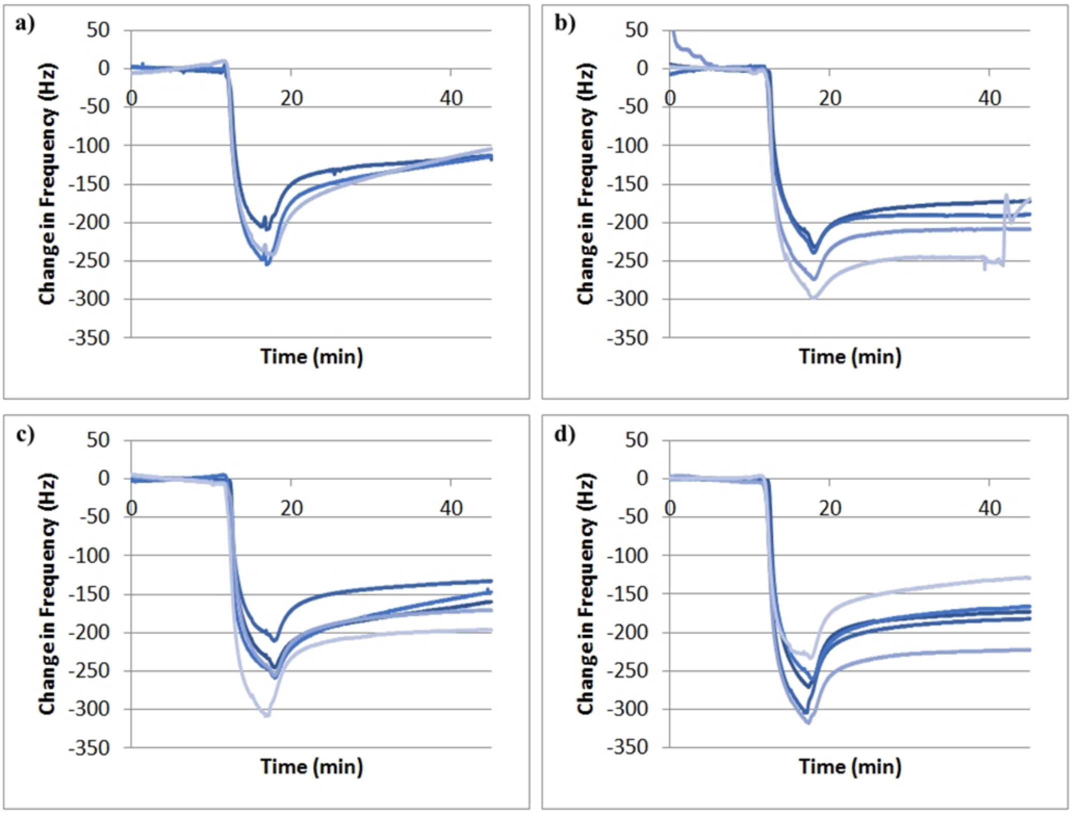

Figure S3. Frequency data for individual TSM runs of PBS buffer with 20% serum containing (**a**) 0 µM LPA, (**b**) 25 µM LPA, (**c**) 50 µM LPA, and (**d**) 100 µM LPA.

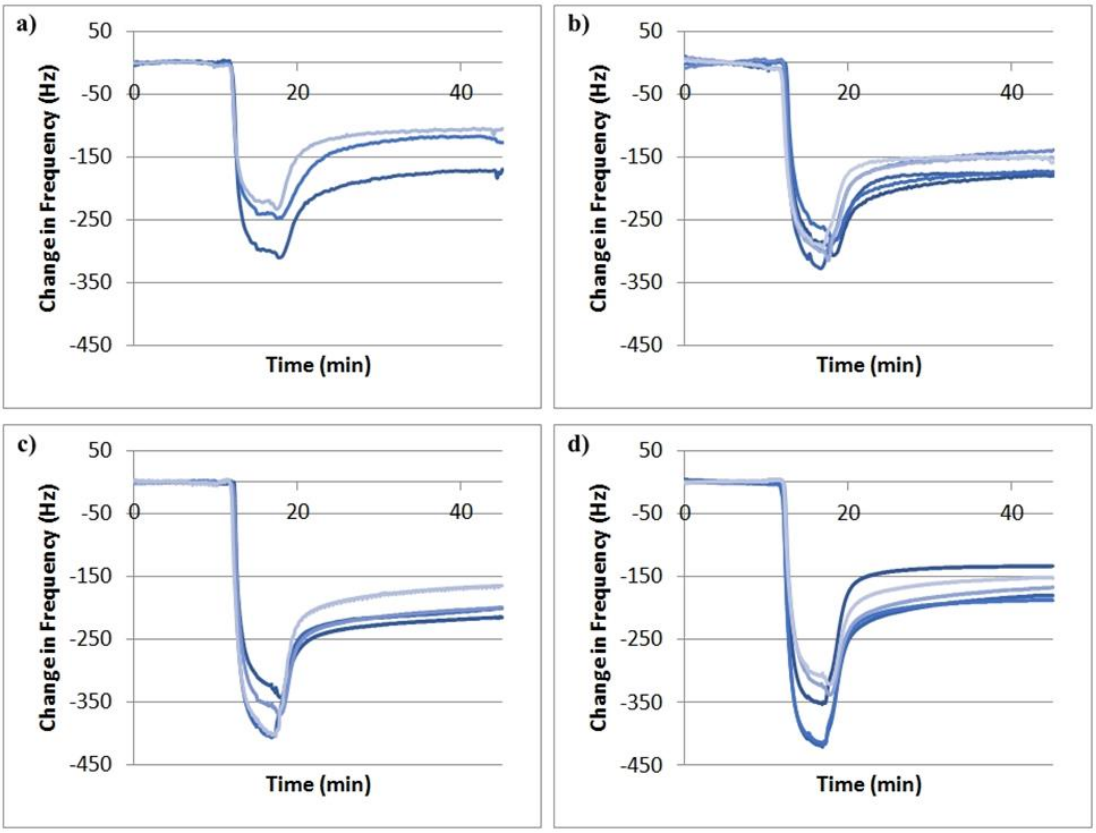

Figure S4. Frequency data for individual TSM runs of PBS buffer with 50% serum containing (**a**) 0 µM LPA, (**b**) 25 µM LPA, (**c**) 50 µM LPA, and (**d**) 100 µM LPA.

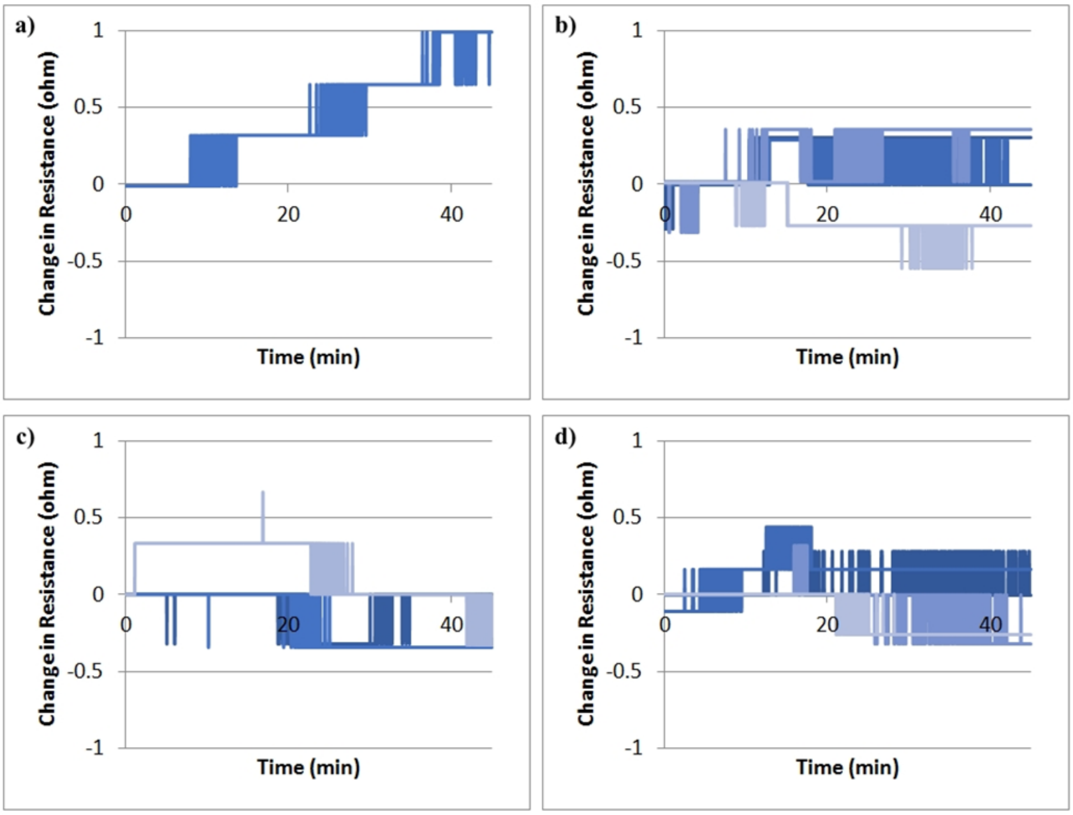

Figure S5. Resistance data for individual TSM runs of 100% PBS buffer containing (**a**) 0 µM LPA, (**b**) 25 µM LPA, (**c**) 50 µM LPA, and (**d**) 100 µM LPA.

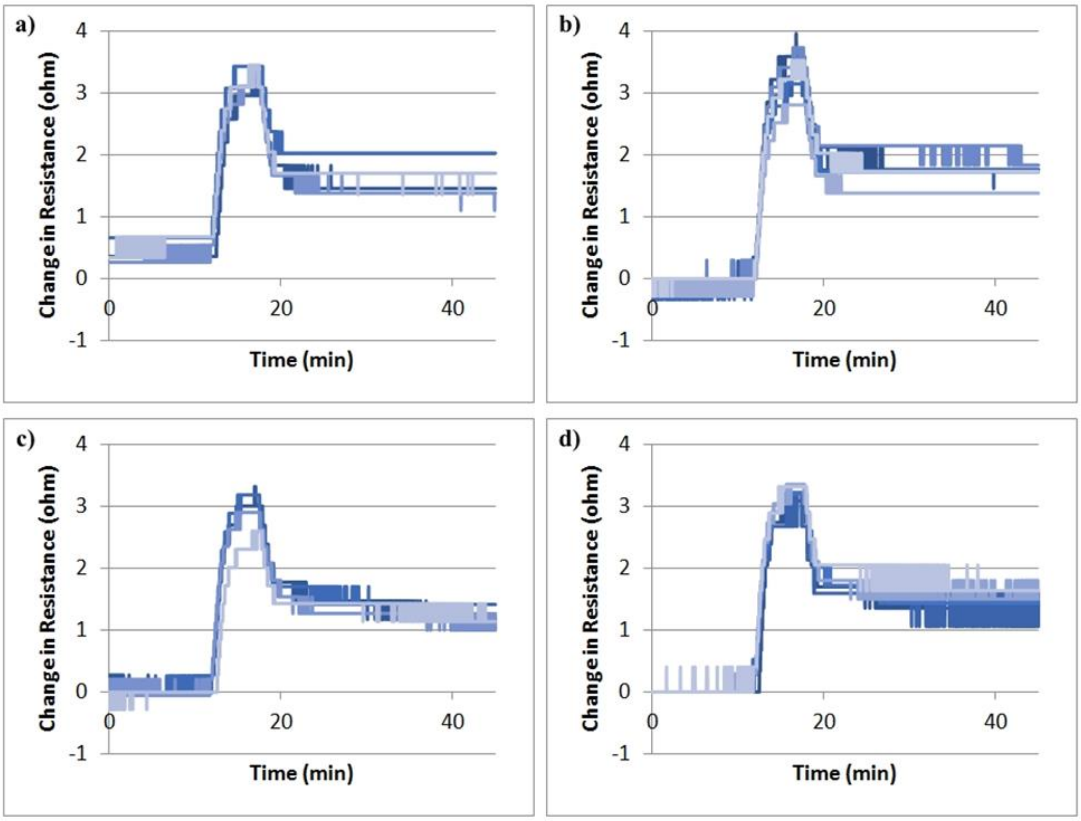

Figure S6. Resistance data for individual TSM runs of PBS buffer with 10% serum containing (**a**) 0 μM LPA, (**b**) 25 μM LPA, (**c**) 50 μM LPA, and (**d**) 100 μM LPA.

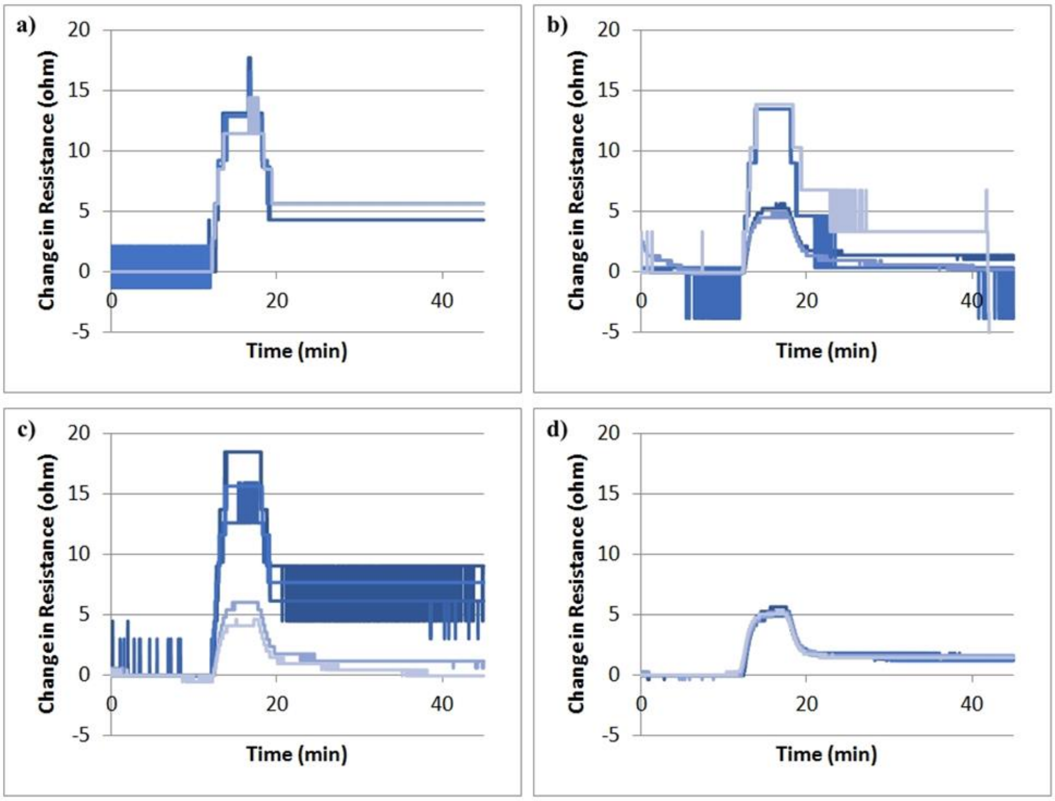

Figure S7. Resistance data for individual TSM runs of PBS buffer with 20% serum containing (**a**) 0 μM LPA, (**b**) 25 μM LPA, (**c**) 50 μM LPA, and (**d**) 100 μM LPA.

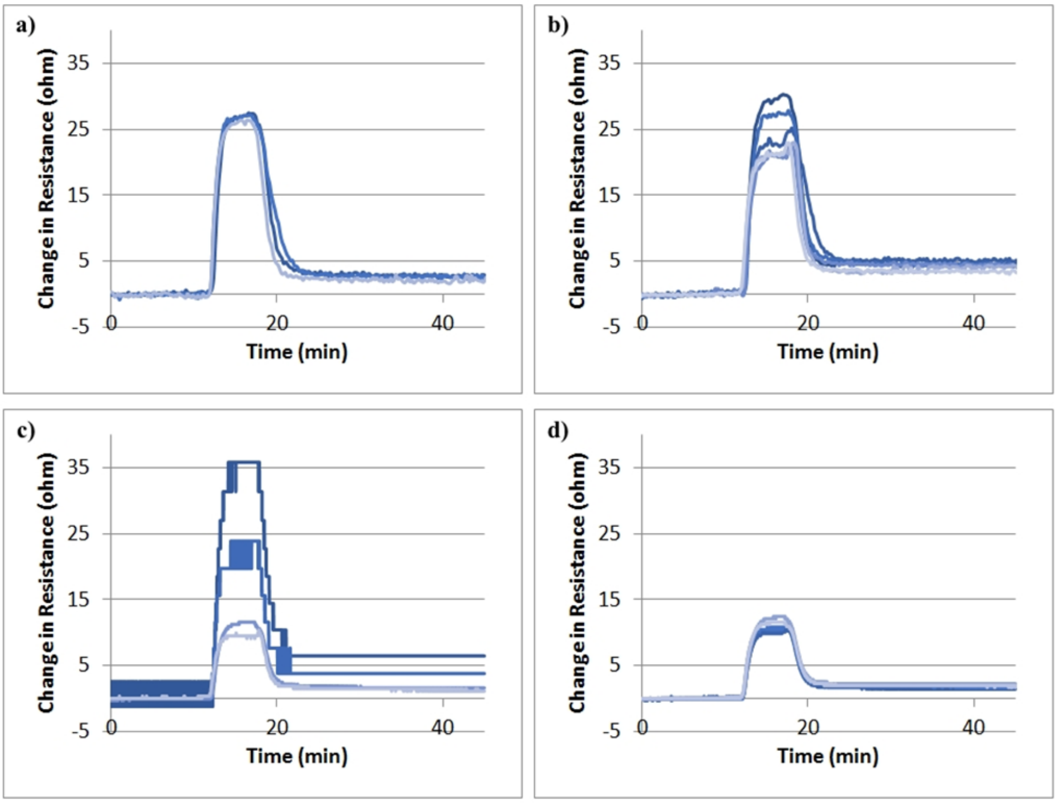

Figure S8. Resistance data for individual TSM runs of PBS buffer with 50% serum containing (**a**) 0 µM LPA, (**b**) 25 µM LPA, (**c**) 50 µM LPA, and (**d**) 100 µM LPA.
